# Gene Turnover Contributes to the Evolutionary Adaptation of *Acidithiobacillus caldus*: Insights from Comparative Genomics

**DOI:** 10.3389/fmicb.2016.01960

**Published:** 2016-12-06

**Authors:** Xian Zhang, Xueduan Liu, Qiang He, Weiling Dong, Xiaoxia Zhang, Fenliang Fan, Deliang Peng, Wenkun Huang, Huaqun Yin

**Affiliations:** ^1^School of Minerals Processing and Bioengineering, Central South UniversityChangsha, China; ^2^Key Laboratory of Biometallurgy of Ministry of Education, Central South UniversityChangsha, China; ^3^Department of Civil and Environmental Engineering, the University of Tennessee, KnoxvilleTN, USA; ^4^Institute of Agricultural Resources and Regional Planning, Chinese Academy of Agricultural SciencesBeijing, China; ^5^Key Laboratory of Plant Nutrition and Fertilizer, Chinese Academy of Agricultural SciencesBeijing, China; ^6^State Key Laboratory for Biology of Plant Diseases and Insect Pests, Institute of Plant Protection, Chinese Academy of Agricultural SciencesBeijing, China

**Keywords:** *Acidithiobacillus caldus*, comparative genomics, intraspecific diversity, gene turnover, evolutionary adaptation

## Abstract

*Acidithiobacillus caldus* is an extremely acidophilic sulfur-oxidizer with specialized characteristics, such as tolerance to low pH and heavy metal resistance. To gain novel insights into its genetic complexity, we chosen six *A. caldus* strains for comparative survey. All strains analyzed in this study differ in geographic origins as well as in ecological preferences. Based on phylogenomic analysis, we clustered the six *A. caldus* strains isolated from various ecological niches into two groups: group 1 strains with smaller genomes and group 2 strains with larger genomes. We found no obvious intraspecific divergence with respect to predicted genes that are related to central metabolism and stress management strategies between these two groups. Although numerous highly homogeneous genes were observed, high genetic diversity was also detected. Preliminary inspection provided a first glimpse of the potential correlation between intraspecific diversity at the genome level and environmental variation, especially geochemical conditions. Evolutionary genetic analyses further showed evidence that the difference in environmental conditions might be a crucial factor to drive the divergent evolution of *A. caldus* species. We identified a diverse pool of mobile genetic elements including insertion sequences and genomic islands, which suggests a high frequency of genetic exchange in these harsh habitats. Comprehensive analysis revealed that gene gains and losses were both dominant evolutionary forces that directed the genomic diversification of *A. caldus* species. For instance, horizontal gene transfer and gene duplication events in group 2 strains might contribute to an increase in microbial DNA content and novel functions. Moreover, genomes undergo extensive changes in group 1 strains such as removal of potential non-functional DNA, which results in the formation of compact and streamlined genomes. Taken together, the findings presented herein show highly frequent gene turnover of *A. caldus* species that inhabit extremely acidic environments, and shed new light on the contribution of gene turnover to the evolutionary adaptation of acidophiles.

## Introduction

*Acidithiobacillus caldus* (formerly *Thiobacillus caldus*), a moderately thermophilic, obligately chemolithoautotrophic, and extremely acidophilic sulfur-oxidizing bacterium (Hallberg and Lindström, 1994, 1996), is of interest for its potential role in industrial bioleaching (Rawlings, 1998; Dopson and Lindström, 1999). *A. caldus* exploits elemental sulfur and a wide range of reduced inorganic sulfur compounds at moderately high temperatures to support autotrophic growth (Mangold et al., 2011; Chen et al., 2012). It is the primary member of a consortium of sulfur oxidizers in different toxic-laden acidic environments, which are termed “extreme environments,” including coal pile and spoil, gold-bearing reactor operation, as well as low-grade copper bioleaching heap (Valdes et al., 2009; You et al., 2011; Zhang et al., 2016c). Considering that *A. caldus* inhabits harsh environments for prolonged periods and accommodates both sudden stress changes and long-term stress conditions in various habitats, gene flow and genetic drift might frequently occur. As such, the flexible gene repertoire generated by gene exchange has imparted *A. caldus* with extensive genetic material for diversification of function and phenotype. Therefore, research focusing on the correlation between genomic changes and evolutionary adaptation is of great interest.

The accumulation of genomic changes underlying evolutionary adaptation has often been viewed as a complex process, and has been subject to many influences and complications (Barrick et al., 2009). As stated by Carretero-Paulet et al. (2015), homologous genes derived from newly formed subgenomes might undergo asymmetric fractionation via mutational events, which include nucleotide substitutions, gene gains and losses, and changes in genomic structure and organization (Librado et al., 2014). In terms of neutral mutation theory, mutations underlying gene and genome evolution, though not necessarily beneficial, should accumulate at a constant rate by drift (Kimura, 1984). Another view is that the substitution rates for beneficial and deleterious mutations depend on environmental selection, as well as population size and structure (Gillespie, 1991; Ohta, 1992). For many years, the crucial role of gene and genome duplications (namely, neofunctionalization and subfunctionalization) in governing organismal evolution has been acknowledged (Ohno, 1970; Force et al., 1999; Innan and Kondrashov, 2010; Kulmuni et al., 2013). Only in recent decades has great attention been paid to the molecular mechanisms of gene loss (deletion or pseudogenization) as a pervasive source of genetic change, which is believed to be another key evolutionary event that causes adaptive phenotypic diversity (Albalat and Cañestro, 2016). In recent years, a number of analytical methods for population genomics and molecular evolution have provided substantial evidence to determine the relative contribution of diverse evolutionary forces, which shape genome organization, architecture, and diversity in response to environmental perturbations (Librado et al., 2014). In eukaryotes, gene family evolution has often been modeled after a phylogenetic birth-and-death (BD) process (Nei and Rooney, 2005). This BD model, though suitable to account for single-gene duplications, might not be appropriate for calculating gene turnover rates given that horizontal gene transfer (HGT) events occur in certain organisms (Librado et al., 2014). However, an alternative gain-and-death (GD) stochastic model in a maximum-likelihood statistical framework was applied to circumvent this limitation (Librado et al., 2012). Unlike the birth process, gains in the developed GD model can accommodate all kinds of gene acquisitions, irrespective of their original source, even including HGT (Librado et al., 2014). In this study, we are interested in whether the aforementioned theoretical and analytical approaches can be applied to explain the relationship between genetic change and adaptive evolution of *A. caldus* inhabiting extraordinarily extreme environments.

Members of *A. caldus* species are ubiquitous throughout many sulfur-rich acidic environments worldwide (**Table 1**), indicating their adaptation to various niches with high concentrations of toxic substrates, such as coal spoil, gold-bearing bioleaching reactor, and copper mine tailing. In recent years, revolutionary technologies and tools have allowed for the rapid characterization of microbial genome sequences (MacLean et al., 2009; Metzker, 2010). Accurate analyses of gene family evolution have been made possible owing to the increasing availability of closely related genomes (Hahn et al., 2007; Sánchez-Gracia et al., 2009; Vieira and Rozas, 2011). Furthermore, acquisition of numerous additional genomes has fuelled a new field termed comparative genomics (Jacobsen et al., 2011), which is useful for investigating microbial genome evolution and even mechanisms for speciation (González et al., 2014; Justice et al., 2014; Ullrich et al., 2016). Comparative surveys based on the available genomes of the two *A. caldus* strains ATCC 51756 and SM-1 have revealed that both strains harbor a relatively high proportion of unique gene complements (Acuña et al., 2013). These gene complements represent a diverse pool of mobile genetic elements, including insertion sequences (ISs), genomic islands (GIs), and integrative conjugative and mobilizable elements. Yet, limited information is available on the contribution of diverse evolutionary forces to the genomic diversification of *A. caldus*. Given this knowledge gap, we have isolated and sequenced four new *A. caldus* strains from different geographic origins (**Table 1**).

**Table 1:** General features of sequenced chromosomes in *A. caldus* strains.

Organism	*A. caldus* SM-1	*A. caldus* ATCC 51756	*A. caldus* S1	*A. caldus* DX	*A. caldus* ZBY	*A. caldus* ZJ
Geographic origin	Gold-bearing bioleaching reactor, China	Coal spoil at the Kingsbury Mine, UK	Coal heap drainage, Jiangxi, China	Copper mine tailings, Jiangxi, China	Copper mine tailings, Chambishi, Zambia	Copper mine tailings, Fujian, China
Status	Complete	Complete	Draft	Draft	Draft	Draft
Accession number	NC_015850	NZ_CP005986	LZYH00000000	LZYE00000000	LZYF00000000	LZYG00000000
Total bases (bp)	2,932,225	2,777,717	2,792,792	3,122,206	3,160,074	3,143,077
Completeness^∗^			89.75	98.76	98.76	98.14
Coverage	38×	120×	92×	95×	89×	76×
GC content (%)	61.32	61.72	60.90	61.01	60.98	61.00
Number of contigs	1	1	1,208	390	414	386
Maximum sequence length	2,932,225	2,777,717	26,396	102,019	77,380	74,790
Minimum sequence length	2,932,225	2,777,717	200	207	201	206
N50 (bp)	2,932,225	2,777,717	4,735	22,157	18,983	18,308
N90 (bp)	2,932,225	2,777,717	617	4,321	4,123	2,291
Number of rRNA operon (5s-16s-23s)	2	2	1	1	1	1
Number of tRNA	47	49	32	46	47	46
Number of coding sequences	2,833	2,699	2,874	2,942	3,017	2,984
Proteins with predicted function	2,042	2,008	1,860	2,109	2,161	2,144
Reference	You et al. (2011)	Valdes et al. (2009)	This study	This study	This study	This study

*^∗^Genome completeness was estimated using the CheckM. Strains SM-1 and ATCC 51756 with complete genome were excluded.*

In this study, we estimated the phylogenetic relationships of *A. caldus* strains based on their genomic sequences (four newly sequenced genomes and two existing genomes from a public database), and performed an exhaustive study of the GD dynamics, with special focus on genetic exchange underlying evolutionary adaptation. These findings, to some extent, highlight the role of gene turnover in the evolutionary diversification of *A. caldus* and adaptation to specific lifestyles and environmental niches.

## Materials and Methods

### DNA Sequencing and Bioinformatics Analysis

Genome sequences for six strains were retrieved in this study, including *A. caldus* ATCC 51756, SM-1, DX, S1, ZBY, and ZJ. Of these bacteria, the type strain ATCC 51756 was isolated from a coal spoil in Kingsbury, UK (Marsh and Norris, 1983), strain SM-1 was from an industrial reactor used in bioleaching operation (Liu et al., 2007), and the other strains (DX, S1, ZBY, and ZJ) were obtained from the China Center for Type Culture Collection. More details for geographic origins of these four new strains were shown in **Table 1**. Genome sequences of strains ATCC 51756 and SM-1, including chromosomal and plasmid sequences, were downloaded from the GenBank database. For strains DX, S1, ZBY, and ZJ, chromosomal DNA was sequenced by an Illumina MiSeq sequencer (Illumina, Inc., USA), using the paired-end sequencing approach with an average DNA insert size of 300 bp and typical read-length of 150 bp. Subsequently, bioinformatics analysis of raw sequences was performed as described previously (Yin et al., 2014), primarily including quality control, genome assembly, computational prediction of coding sequences (CDS) and other genome features such as rRNA and tRNA, as well as functional assignments against public databases (NCBI-nr and COG). Genome completeness of each strain was also estimated using the program CheckM (Parks et al., 2015). Additionally, circular maps showing chromosome architecture were drawn using the Circos software (Krzywinski et al., 2009).

Intergenomic distance scores were calculated using the web service Genome-to-Genome Distance Calculator (GGDC) 2.1 (Meier-Kolthoff et al., 2013). The distance *d*(*X, Y*) between genome X and Y was calculated according to the formula:

(1) $$d\left(X,Y\right)=1-\frac{2\cdot I_{XY}}{H_{XY}+H_{YX}}$$

in which, *I_XY_* denotes the sum of identical base pairs over all high-scoring segment pairs (HSPs, which are intergenomic matches), while *H_XY_* and/or *H_Y X_* denote the total length of all HSPs. Heatmap was shown using the software HemI (Deng et al., 2014).

### 16S Ribosomal RNA (rRNA) Gene-Based and Whole Genome-Based Phylogenetic Tree

Phylogenetic relationship based on 16S rRNA sequences of *Acidithiobacillus* strains was analyzed using MEGA v5.05 with neighbor-joining method. The robustness of clustering was evaluated by 1,000 bootstrap replicates. Additionally, the phylogenetic relationships between complete and draft genomes from *A. caldus* strains were estimated. We employed an online platform CVTree3 (Zuo and Hao, 2015) to construct the whole-genome based phylogenetic tree using a composition vector approach. This whole-genome-based and alignment-free prokaryotic phylogeny was validated by directly comparing our result with the taxonomy of these strains, as opposed to performing statistical resampling tests such as bootstrap or jackknife. The genome sequence of *Acidithiobacillus ferrooxidans* ATCC 23270 was chosen as an outgroup. Subsequently, visualization of phylogenetic tree was executed using the MEGA v5.05 (Tamura et al., 2011).

### Pan-Genome Analysis

Species diversity could be identified by analyzing gene repertoire across all strains of a species, i.e., the pan-genome (Tettelin et al., 2008). PanOCT v3.18 (Fouts et al., 2012) with a BLASTP all-against-all comparison of entire proteins (*E*-value ≤ 1e^-5^; sequence identity ≥ 50%) was used to identify shared and unique gene content. Subsequently, annotation of core genome and strain-specific genes was implemented using BLAST against the extended COG database (Franceschini et al., 2013).

### Gene Family Evolution

Groups of orthologous sequences (orthogroups, herein referred to as gene families) in all six *A. caldus* strains were classified by clustering with OrthoFinder v0.4 (Emms and Kelly, 2015), using a Markov cluster algorithm. Transposable elements were excluded, given that these gene sequences might interfere with our analyses owing to lineage-specific expansions (Carretero-Paulet et al., 2015).

To analyze the evolutionary rates of gene families, we applied the developed computational program BadiRate v1.35 using a GD stochastic model (Librado et al., 2012). The gain (γ) and death (δ) rates of gene families were estimated using a branch-specific rates (GD-BR-ML) model assuming that each phylogenetic branch had its own specific turnover rate.

### Mobile Gene Elements, Insertion Sequence Elements, Transposable Elements, and Genomic Islands

IS family annotation and transposase inspection was done by BLAST comparison (*E*-value ≤ 1e^-5^) against the ISFinder database with manual detection of the surrounding significant search hits (Siguier et al., 2006). The program SeqWord Genomic Island Sniffer (Bezuidt et al., 2009) was implemented to identify the putative horizontally transferred elements distributed in the chromosome of *A. caldus* ATCC 51756. Then, the prediction of genes in the putative horizontally transferred elements was performed using the MetaGeneAnnotator (Noguchi et al., 2008). For the other chromosomes, the computational tool IslandViewer 3 (Dhillon et al., 2015), which integrates three different prediction methods including IslandPick (Langille et al., 2008), IslandPath-DIMOB (Hsiao et al., 2003), and SIGI-HMM (Waack et al., 2006), was used to predict GIs. The GC content of GI sequences was calculated using the NGS QC Toolkit (Patel and Jain, 2012). Due to the high number of contigs, *A. caldus* S1 was excluded from the GI prediction.

### Availability of Supporting Data

The data sets supporting our results in this study are available in the GenBank repository. These Whole Genome Shotgun projects of four newly sequenced *A. caldus* strains have been deposited at the DDBJ/ENA/GenBank under the accession numbers LZYE00000000 (DX), LZYF00000000 (ZBY), LZYH00000000 (S1), and LZYG00000000 (ZJ). Additionally, the versions described in this paper are version LZYE01000000, LZYF01000000, LZYH01000000, and LZYG01000000, respectively.

## Results and Discussion

### Overview of the *A. caldus* Chromosomes

The circular chromosomes of *A. caldus* strains varied from 2.78 to 3.16 Mb (**Table 1**). *A. caldus* strains DX, ZBY, and ZJ, which were isolated from a copper mine, possess larger chromosomes than the other strains inhabiting the divergent habitats. Genome-size variations in bacteria correspond to variations in gene number as bacterial genomes are tightly packed, and most sequences are functional protein-coding regions (Mira et al., 2001). Accordingly, strains with larger genome were predicted to harbor more CDSs compared to other strains in this study. Additionally, the evaluation of quality and completeness of genome assemblies supported the reliability of pan-genome analysis, although strain S1 had relatively low genome completeness in comparison with its closely related counterparts (**Table 1**).

In all *A. caldus* strains, the mean percentage GC content of these chromosomal DNAs (60.90–61.72% for all six strains) was much higher than that observed for other recognized *Acidithiobacillus* spp., e.g., *A. ferrooxidans, A. thiooxidans*, and *A. ferrivorans*. It might be reasonable considering that *A. caldus* species was known as the only known mesothermophile within the Acidithiobacillales (Acuña et al., 2013), and GC content of prokaryotic genomes was positively correlated with optimal growth temperature (Musto et al., 2004, 2006).

### Evolutionary Relationship of *A. caldus* Strains

**FIGURE 1:**
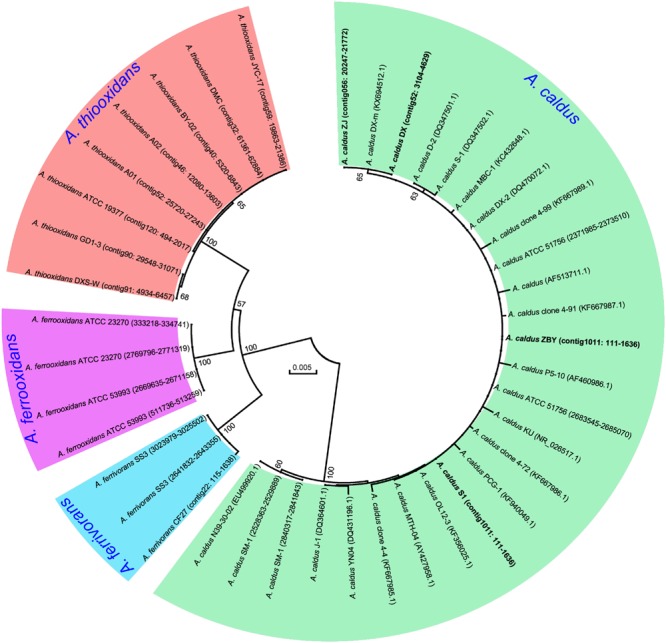
**Phylogenetic tree of 16S rRNA genes showing the relationship between newly sequenced strains and other *Acidithiobacillus* strains.** The accession numbers of gene sequences or genomic loci are given in parentheses. Four new strains in this study are highlighted in bold.

**FIGURE 2:**
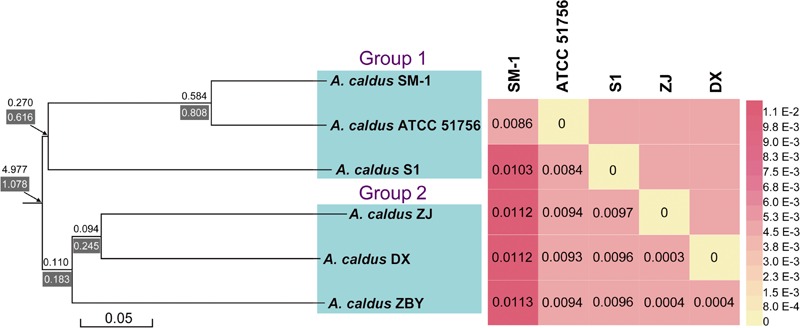
**A collective diagram showing phylogenetic relationships, the genome-content-based distance matrix, and gene turnover rates.** The phylogenetic tree depicts the relationships of currently sequenced *A. caldus* strains using a composition vector approach, and genome-to-genome distances were visualized using the heatmap. Additionally, values on/below each phylogenetic branch indicate the gene gain/death rate, respectively.

A phylogenetic tree based on 16S rRNA genes of *Acidithiobacillus* strains preliminarily demonstrated that these four newly sequenced strains in this study were taxonomically affiliated with *A. caldus* (**Figure 1**). To further identify the evolutionary relationships of *A. caldus* strains, an whole-genome-based and alignment-free phylogenetic tree was constructed (**Figure 2**). Additionally, GGDC analyses were employed to support the phylogenetic relationship. This phylogenomic tree showed that three strains isolated from the copper mine (namely, ZJ, DX, and ZBY) were clustered together (group 2 in **Figure 2**). Similarly, an earlier study reported that taxonomic clustering of six strains belonging to the genus *Novosphingobium* was generally influenced by their respective source of isolation (Gan et al., 2013). Further inspection revealed that the geographic distribution of strain ZBY was distinctively differed from those of the other two strains (ZJ and DX), and the genome-content-based distance matrix implied a slight evolutionary divergence (**Figure 2**). The correlation between intraspecific divergence and geographic distribution was also observed within the closely related *A. thiooxidans* species by comparative genomic analysis (Zhang et al., 2016a). In the group 1 (**Figure 2**), interestingly, *A. caldus* SM-1 was obtained from a bioleaching reactor used for low grade gold-bearing minerals (Acuña et al., 2013), and the strain ATCC 51756 was isolated from a coal spoil; moreover, phylogenetic analysis revealed that these two strains were more closely related to each other than to the other four strains examined in this study. We therefore suspect that strain SM-1 might originally be isolated from an acidic setting similar to the habitat for ATCC 51756.

Ji et al. (2014) showed that the differences in adaptive evolution were attributable to different econiche by genetically analyzing the marine and freshwater magnetospirilla. Accordingly, we propose that environmental variation, particularly geochemical conditions, might be a determinant of genomic diversity of *A. caldus* strains. From an alternative perspective, it appears that geographic distribution has less of an influence on hereditary variation in comparison with econiche difference. The findings were consistent with an earlier study showing that environmental heterogeneity has relatively more influence on microbial biogeography compared to geographic distance (Lin et al., 2013).

### Gene Contents in *A. caldus* Strains

Gene prediction showed that the chromosomes of *A. caldus* strains contained 2,699 (ATCC 51756), 2,833 (SM-1), 2,874 (S1), 2,942 (DX), 3,017 (ZBY), and 2,984 (ZJ) predicted CDS. Functional analysis based on COG categories (Supplementary Table S1) revealed that the four most abundant functional categories within all *A. caldus* strains were “function unknown [S],” “replication, recombination, and repair [L],” “cell wall/membrane/envelope biogenesis [M],” and “energy production and conversion [C].” As reported by Silver and Phung (1996), high concentrations of toxic substrates such as heavy metals might cause a high rate of DNA damage. Thus, it was expected that CDS involved in COG category [L] would be abundant in *A. caldus* strains. Additionally, these data can also explain why this finding was distinct from previous studies analyzing the COG classification of other organisms such as marine magnetospirillum *Magnetospira* sp. QH-2 (Ji et al., 2014), given that the concentrations of potential toxic substrates in the extreme environment were much higher than those in the marine environment.

A previous study based on four genomes of “*Ferrovum*” strains highlighted the most distinct differences in interspecific metabolisms (Ullrich et al., 2016). However, in our study the assignment of CDS to the COG classification revealed that no significant differences in the number of assigned CDS were observed between the six genomes (Supplementary Table S1), probably suggesting few group-specific metabolic traits.

### Comparison of Inferred Metabolic Traits and Niche Adaptation

#### Comparison of the Central Metabolism

**FIGURE 3:**
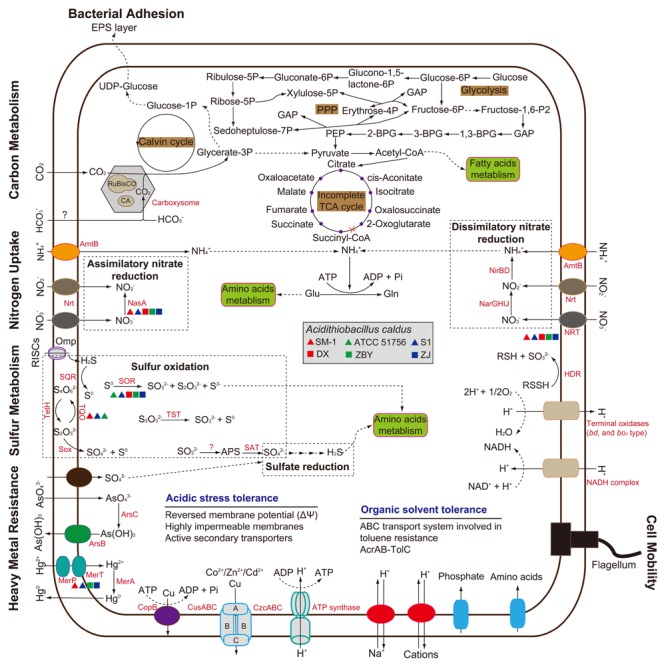
**Genome-guided model for the central metabolism and niche adaptation of *A. caldus* strains.** This whole-cell model was adapted from several previous studies (Justice et al., 2014; Ullrich et al., 2016; Zhang et al., 2016b). Predicted genes involved in cellular metabolism and stress management are listed in Supplementary Table S2.

In light of COG assignment aforementioned, we further observed CDS related to the predicted metabolic profiles. Compared with other metabolic models reported in the literature, including carbon metabolism (You et al., 2011; Zhang et al., 2016b), nitrogen uptake (Levicán et al., 2008; Justice et al., 2014), and sulfur oxidation (Mangold et al., 2011; Chen et al., 2012; Yin et al., 2014), all strains in our study were predicted to contain numerous genes involved in central metabolism (Supplementary Table S2). The metabolic potentials of all strains were reconstructed and compared to each other for the identification of shared metabolic features as well as group- or strain-specific traits (**Figure 3**). Comprehensive analysis of these metabolism-related genes focuses on the main differences between the six *A. caldus* strains. As depicted in **Figure 3**, however, the evidence showed low intraspecific genetic diversity in the predicted metabolic profiles between *A. caldus* strains. A suite of genes involved in carbon assimilation were found in all strains. *A. caldus* fixes carbon dioxide via the classical Calvin–Benson–Bassham (CBB) cycle, and harbors a gene cluster predicted to encode carbon dioxide-concentrating protein (CcmK) with various copies, carboxysome shell protein (CsoS), carboxysomal shell carbonic anhydrase (CsoSCA), and ribulose-1,5-bisphosphate carboxylase/oxygenase (RuBisCO; Supplementary Table S2). Moreover, *A. caldus* operates a complete Embden–Meyerhof pathway (EMP) or glycolysis, pentose phosphate pathway (PPP), and incomplete tricarboxylic acid (TCA) cycle, which lacks the 2-oxoglutarate dehydrogenase complex (Valdés et al., 2008b).

With respect to nitrogen uptake, although *A. caldus* lacks nitrogenase directing the fixation of molecular nitrogen (Valdés et al., 2008a), assimilation of nitrate, nitrite, and ammonia plays a critical role in meeting nitrogen requirements. *A. caldus* utilizes nitrate or nitrite via nitrate transporter (NRT) and nitrate/nitrite transporter (Nrt). However, NRT was not present in strain ATCC 51756. Genes associated with dissimilatory nitrate reduction were identified, while a gene involved in assimilatory nitrate reduction (*nasA*) was absent in strain ATCC 51756. Though absent, it appears that the non-existence of those genes had little influence on the assimilation of nitrate. Additionally, all strains share the potential to take up extracellular ammonia into the cell via AmtB transporter (**Figure 3**) under low nitrogen levels (Levicán et al., 2008), and to convert it to glutamine via glutamine synthetase.

In recent years, the sulfur oxidation system in *A. caldus* has been well studied (Mangold et al., 2011; Chen et al., 2012). According to reported sequences, numerous genes related to sulfur oxidation were found. Additionally, all *A. caldus* strains harbor genes predicted to be involved in sulfate reduction (Supplementary Table S2). Of note, the *sor* gene encoding sulfur oxygenase reductase, an important enzyme catalyzing a disproportionation reaction of cytoplasmic sulfur (Zhang et al., 2015), was absent in strain SM-1 (**Figure 3**). Group 2 strains lack the gene encoding the putative thiosulfate:quinone oxidoreductase. Thus, whether other alternative genes exist in these strains needs to be studied further. Similar to the well-studied model for electron transfer of *A. ferrooxidans* (Valdés et al., 2008a), *A. caldus* potentially employs the electron transfer pathway from sulfur oxidation to (1) various types of terminal oxidases to generate a proton gradient or (2) to NADH complex to produce reducing power (Chen et al., 2012).

To some extent, investigation of genes involved in central metabolism supported the results of COG assignment that there were no obvious intraspecific differences. In other words, comparison of intraspecific genomes showed that only slight differences were observed in metabolic profiles, at least in central metabolism.

#### Response to Environmental Stress

Microbial response to environmental stresses is always a critical issue in ecological fields (Yin et al., 2015). A long-term experiment with *Escherichia coli* revealed complex coupling between organismal adaptation and genome evolution, which occurred even in a constant environment (Barrick et al., 2009). In the context of the six *A. caldus* strains, bacterial adhesion, motility, heavy metal resistance, and organic solvent tolerance were taken into account (**Figure 3**). All strains share a core set of genes potentially related to environmental adaptation (Supplementary Table S2). The presence of genes encoding extracellular polymeric substances precursors and type IV pili in *A. caldus* suggests a cell adhesion on mineral surface. This trait provides a reaction space between cell and mineral surface, thereby increasing the dissolution of metal sulfides (Watling, 2006; González et al., 2013). Genes assigned to COG category [N] (cell mobility) and [T] (signal transduction) were also observed, but there were few differences between these two groups (Supplementary Table S1). A full suite of genes associated with flagellar assembly were found in all strains, suggesting that *A. caldus* strains had the capacity to swim across environmental gradients and to colonize new sites.

Extremely acidic environments s, especially bioleaching systems, are regarded as having extremely high concentrations of soluble and potentially toxic substrates such as heavy metals, including arsenic, mercury, copper, and cadmium (Valdés et al., 2008a) and organic extractants, such as Lix984n (Zhou et al., 2012). A series of gene clusters potentially encoding functional enzymes were identified, suggesting that *A. caldus* has the ability to cope with high concentrations of heavy metal ions. As for organic solvent tolerance, a six-gene cluster, encoding ABC transporter ATP-binding protein, hypothetical protein, toluene tolerance protein, mce-related protein, toluene tolerance protein Ttg2B, and toluene ABC transporter ATP-binding protein, was found in all strains. Additionally, an *acrAB*-*tolC* operon potentially encoding AcrB (transporter AcrB/AcrD/AcrF family protein), AcrA (RND family eﬄux transporter MFP subunit), and TolC (outer membrane eﬄux protein) in each genome indicated that *A. caldus* can utilize the pumps associated with resistance-nodulation-cell division protein to transfer these organic substrates.

### Pan-Genome Analysis

**FIGURE 4:**
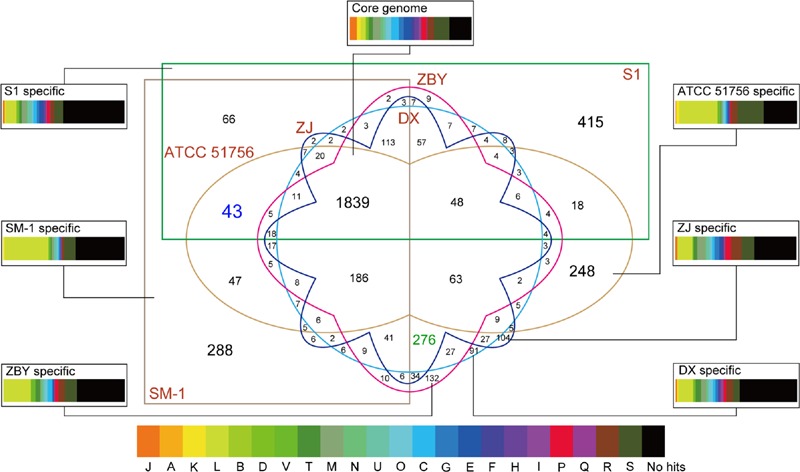
**Six-way Venn diagram of the pan-genome of *A. caldus* species.** Various shapes and colors indicate different strains. Numbers of core genome as well as accessory genome in given patterns are shown in the Venn diagram. Number with blue color indicates genes shared by group 1 strains, while number with green color represents genes shared by group 2 strains. Specially, the core genome and strain-specific genes were used to search against the COG database. The colored rectangles with various widths represented the proportion of CDSs related to COG categories.

As shown above, numerous homologous genes associated with metabolic pathways as well as environmental adaptation were observed. To gain a deeper understanding of group- and strain-specific features, pan-genome analysis of *A. caldus* species was performed. A total of 4,424 CDS acquired from the four newly sequenced chromosomes plus two available chromosomes in the public database were clustered using the PanOCT. Pairwise BLAST comparisons indicated that 1,839 orthologs (41.57%) with a high percentage across all six strains were identified as the *A. caldus* core genome (**Figure 4**). The remaining variable 1,307 clusters were classified as the *A. caldus* accessory genome. Furthermore, strain-specific clusters were observed among the six *A. caldus* strains.

Functional assignment based on the core genome was employed to investigate the proportion of proteins in each COG category. As depicted in **Figure 4**, the core genome in *A. caldus* strains was commonly enriched in the COG category [M] (cell wall/membrane/envelope biogenesis; 6.36%). Additionally, our results showed that CDSs involving COG categories [C] (energy production and conversion; 6.30%) and [E] (amino acid transport and metabolism; 6.25%) were abundant. The large proportion of these genes indicated that energy utilization and uptake of nutrients in these strains might be more efficient to better adapt to the challenging environment. In other words, these findings were in line with an earlier report detailing that core genes provided functions that were essential to the basic lifestyle of the species (Medini et al., 2005).

Persistent genes encoding essential functions are stably maintained in genomes under constant selection (Nuñez et al., 2013), while dispensable or accessory genes are frequently gained or lost (Medini et al., 2005). Therefore, the accessory genome contributes to intraspecific diversity (Tettelin et al., 2008). Here, we identified many transposases by alignment of accessory genes against the NCBI-nr database (Supplementary Table S3), suggesting roles in shaping the evolution of protein families. Similarly, previously studies based on available genomes revealed that plentiful accessory genes were probably acquired by HGT (Tian et al., 2012; Sugawara et al., 2013). Additionally, it is particularly noteworthy that strain-specific genes were found to be enriched in the COG category [L] (replication, recombination and repair; **Figure 4**), thus supporting the view that the accessory genome confers selective advantages such as niche adaptation.

In particular, a total of 43 and 276 group-specific genes shared by group 1 and group 2 strains, respectively, were detected (**Figure 4**). Functional profiling based on COGs revealed that most of these predicted CDS were assigned to no COG category, probably indicating the existence of many group-specific CDS with unidentified function (Supplementary Table S4). Further inspection underscored that the abundant genes involved in certain COG categories, including [L] (replication, recombination, and repair), [M] (cell wall/membrane/envelope biogenesis), and [P] (inorganic ion transport and metabolism), might be necessary for the group 2 strains. A reasonable explanation is that copper bioleaching heap, the habitat for group 2 strains, has high concentrations of toxic metals (Zhang et al., 2016c). Microbes in such an extreme environment might harbor potential strategies to cope with the chemical constraints of their natural functions. Additionally, COG categories [S] (general function prediction only) and [R] (function unknown) were relatively abundant in all groups, further highlighting the role of these unknown functional CDSs in genomic differentiation.

### Mobile and Transposable Elements

Prediction and classification of transposable elements using ISFinder indicated that a large number of IS elements, which accounted for various proportions of the total CDS in each chromosome (ranging from 1.8 to 5.8%), were randomly distributed over the chromosomes of the *A. caldus* strains (Supplementary Table S5). Although the types of IS families were similar to each other, their distribution and relative abundance varied with each strain. Among them, some of these IS elements were identified to cluster in flexible chromosomal regions that did not satisfy the criteria of other putative mobile elements such as GIs; these findings were consistent with those from an earlier study (Acuña et al., 2013). As stated by Bentley and Parkhill (2004), the progressive loss of gene order in a prokaryotic genome might be attributed to several events including gene deletion, IS and repeat expansion, as well as recombination or rearrangement. Given this, *A. caldus* SM-1 as well as ATCC 51756 might have higher genome plasticities compared with other closely related strains, mainly because of the acquisition of IS elements during evolution.

Aside from IS elements, the putative GI elements in all *A. caldus* strains were also identified. Results showed that several GIs ranging from 4 to 58 kb were widespread in the chromosomes of *A. caldus* strains (Supplementary Table S6). Additionally, most CDS in the GIs were annotated as hypothetical proteins. Further analyses showed the presence of integrases or mobile genetic elements such as transposase, thereby indicating that various putative GIs might be acquired via HGT. In light of the view that underscores the contribution of horizontal (lateral) gene transfer (HGT) in the expansion of gene repertoires of prokaryotes (Ochman et al., 2000; Gogarten et al., 2002; Treangen and Rocha, 2011), we inferred that the frequency of HGT was high in group 2 strains with larger genomes, conferring a predominant role in shaping their evolution and allowing the acquisition of novel adaptive functions. We emphasized the role of GIs in adaptation to specific lifestyles and environmental niches, considering that many GIs were highly relevant for niche-specific adaptation (Wu et al., 2011). Furthermore, numerous genes in *A. caldus* species might be obtained by genetic exchange as suggested by the presence of a large load of mobile genetic elements including IS elements, transposases, and GIs. Consequently, changes in genome structure and gene copy number might provide *A. caldus* strains with a survival advantage for rapid adaptation and survival in highly acidic and metal-laden environments.

### Probabilistic Analysis of Gene Family Turnover

Gene families in *A. caldus* strains were classified as orthogroups using OrthoFinder (**Table 2**). Our classification identified up to 3,109 orthogroups (containing two or more genes in all selected strains), which included 16,470 sequences. There were fewer genes in group 1 strains with small genomes clustered into multigene orthogroups than in group 2 strains. However, these smaller genomes contained more unassigned genes than any orthogroups compared to the others. These results appear to be explained in part by intense fractionation pressure (Carretero-Paulet et al., 2015). In other words, multigene families in smaller genomes might be under continuous deletion pressure and, as a result, these genomes tend to be smaller in comparison with their counterparts in larger genomes.

**Table 2:** Summary of genes assigned or unassigned to orthogroups in six *A. caldus* strains.

Number	SM-1	ATCC 51756	S1	ZJ	DX	ZBY
Orthogroups	2,514	2,422	2,395	2,818	2,800	2,828
Genes in orthogroups	2,709	2,538	2,477	2,918	2,886	2,942
Genes unassigned to any orthogroup	124	161	397	66	56	75

BadiRate analysis, using a full likelihood method, was applied to examine the evolutionary dynamics of gene families across the *A. caldus* species, and to characterize the expansion and/or contraction of genomes. The statistical framework not only estimates GD rates in a decoupled manner, using two independent parameters (γ and δ), but also explicitly takes into account certain key features in prokaryotic evolution, such as HGT. A stochastic GD-BR-ML model statistically evaluating the turnover rates demonstrated that a large number of orthologous genes frequently undergoing high gain and/or death events have evolved from ancestral genes (**Figure 2**). Particularly, gene families in *A. caldus* species rapidly expand through gene gain (duplication) and slowly contract through gene death (deletion or pseudogenization), indicating that the extensive recruitment of genes involved in long-term evolution confers an ecological advantage for survival and proliferation under extremely acidic conditions. Moreover, the phylogenetic branch with the higher death rate (δ = 0.616 and/or 0.808) indicated that group 1 strains with smaller genomes might be derived from free-living ancestors by the genome-reductive evolutionary process (**Figure 2**). Given that genome reduction coincided with the increase in frequency of mobile elements and repeated sequences (Moran, 2003), multiple IS elements identified in strain SM-1 and ATCC 51756 (Supplementary Table S5) might play a key role in mediating intrachromosomal recombination, thereby leading to rearrangements and gene loss. However, the dispensable genes in the above-mentioned microorganisms might suffer extensive loss and non-functionalization. The compact genomes in the given organisms can perform essential functions for cellular survival and replication, as the loss of dispensable genes has little effect on bacterial fitness, at least under certain environmental conditions (Albalat and Cañestro, 2016). Despite their smaller or near-minimal size, all reduced genomes still retain the essential gene set, and are thereby able to support cellular life both in stable and changing circumstances (Moya et al., 2009). Therefore, small genomes in group 1 strains would be more tightly packed by selective reduction, and are thus more streamlined than their larger genome counterparts.

Gene turnover in group 2 strains was also estimated. As illustrated in **Figure 2**, phylogenetic branches showed a lower gene turnover rate in group 2 strains compared to that in group 1 strains. Additionally, we found that the rates of gene death were slightly higher than the gain turnover rates. There were two possible explanations for these results. The number of genes gained from HGT as well as gene duplication events might be significant enough to account for the increase of microbial DNA content and novel functions, and play a key role in evolution (Mira et al., 2001; Navarre et al., 2006). This hypothesis may also be supported by an earlier genetic study on the evolution of *Bacillus anthracis* virulence, which revealed that key genes that cause anthrax in this bacterium were identified as acquired by HGT (Zwick et al., 2012). However, a conceivable explanation underlying environment-dependent conditional dispensability indicates that genes in a given species would be dispensable if they were related to certain processes that were only required in a specific untested environments (Albalat and Cañestro, 2016). Of note, it is challenging to assess which genes are regarded as dispensable or essential components by coupling genotypes with phenotypes. In view of the complexity of environmental conditions in copper mines, low deletion pressures might provide microbes with a major fitness advantage for growth in adverse environments. Furthermore, large genomes in bacteria correspond to species that have the ability to tackle various environmental stimuli (Schneiker et al., 2007). Accordingly, large bacterial genomes might have an adaptive role in the evolution of group 2 strains.

## Conclusion

Six chromosomes of the extreme acidophile *A. caldus* were valuable resources for the investigation of genetic diversity and evolutionary adaptation. A phylogenetic tree based on chromosomal sequences of *A. caldus* species showed a potential correlation between genomic diversity and geochemical characteristics. Further analysis revealed that chemical constraint in respective natural habitat might be a determinant contributing to genetic diversification. Apparently, genetic analyses indicated that gene gain and loss were both dominant evolutionary forces in the adaptive evolution of *A. caldus* species. During adaptation to these adverse environmental conditions, GD rates varied in different settings, resulting in genomic differentiation and speciation. The compact and streamlined genomes might undergo selective deletion pressure, whereas large genomes had been extensively recruited by intraspecific or interspecific genetic exchange. These genome-guided findings in our study, to some extent, provide novel insights into the evolutionary adaptation of *A. caldus* species.

## Author Contributions

XnZ, XL, and QH conceived and designed the experiments. XnZ and WD performed the experiments. XnZ analyzed the data. XnZ wrote the paper. XoZ, FF, DP, WH, and HY revised the manuscript.

## Conflict of Interest Statement

The authors declare that the research was conducted in the absence of any commercial or financial relationships that could be construed as a potential conflict of interest.
