# Supplementary material for: Gene Turnover Contributes to the Evolutionary Adaptation of Acidithiobacillus caldus: Insights from Comparative Genomics
Source: Front Microbiol. 2016 Dec 6;7:1960. doi: 10.3389/fmicb.2016.01960 (PMC5138436; doi:10.3389/fmicb.2016.01960)
Supplement: Supplementary file 1 [file Table_1.docx]

**Supplementary Table S1** The COG functional assignment of genes from *A. caldus* strains.

| **COG category^a^** | **Group1** | | | | | | **Group2** | | | | | |
| --- | --- | --- | --- | --- | --- | --- | --- | --- | --- | --- | --- | --- |
|  | **SM-1** | | **ATCC 51756** | | **S1** | | **DX** | | **ZBY** | | **ZJ** | |
|  | **Number** | **Percent (%)** | **Number** | **Percent (%)** | **Number** | **Percent (%)** | **Number** | **Percent (%)** | **Number** | **Percent (%)** | **Number** | **Percent (%)** |
| **Information storage and processing** | | | | | | | | | | | | |
| J | 118 | 5.78 | 118 | 5.88 | 130 | 6.46 | 118 | 5.60 | 114 | 5.28 | 116 | 5.41 |
| A | 1 | 0.05 | 1 | 0.05 | 1 | 0.05 | 1 | 0.05 | 1 | 0.05 | 1 | 0.05 |
| K | 72 | 3.53 | 67 | 3.34 | 66 | 3.28 | 75 | 3.56 | 74 | 3.42 | 73 | 3.40 |
| L | **236** | **11.56^b^** | **197** | **9.81^b^** | **151** | **7.51^b^** | **194** | **9.20^b^** | **216** | **10.00^b^** | **199** | **9.28^b^** |
| B | 1 | 0.05 | 1 | 0.05 | 2 | 0.10 | 1 | 0.05 | 1 | 0.05 | 1 | 0.05 |
| **Cellular processes and signaling** | | | | | | | | | | | | |
| D | 29 | 1.42 | 29 | 1.44 | 30 | 1.49 | 33 | 1.56 | 31 | 1.43 | 31 | 1.45 |
| V | 42 | 2.06 | 34 | 1.69 | 42 | 2.09 | 40 | 1.90 | 44 | 2.04 | 40 | 1.87 |
| T | 70 | 3.43 | 73 | 3.64 | 82 | 4.08 | 76 | 3.60 | 79 | 3.66 | 80 | 3.73 |
| M | **152** | **7.44^b^** | **153** | **7.62^b^** | **165** | **8.20^b^** | **165** | **7.82^b^** | **170** | **7.87^b^** | **172** | **8.02^b^** |
| N | 43 | 2.11 | 59 | 2.94 | 44 | 2.19 | 65 | 3.08 | 66 | 3.05 | 63 | 2.94 |
| U | 69 | 3.38 | 54 | 2.69 | 65 | 3.23 | 67 | 3.18 | 69 | 3.19 | 70 | 3.26 |
| O | 98 | 4.80 | 95 | 4.73 | 95 | 4.72 | 107 | 5.07 | 105 | 4.86 | 106 | 4.94 |
| **Metabolism** | | | | | | | | | | | | |
| C | **148** | **7.25^b^** | **143** | **7.12^b^** | **151^b^** | **7.51^b^** | **148** | **7.02^b^** | **154** | **7.13^b^** | **150** | **7.00^b^** |
| G | 80 | 3.92 | 84 | 4.18 | 90 | 4.48 | 84 | 3.98 | 84 | 3.89 | 89 | 4.15 |
| E | 130 | 6.37 | 128 | 6.37 | 135 | 6.71 | 128 | 6.07 | 131 | 6.06 | 132 | 6.16 |
| F | 51 | 2.50 | 53 | 2.64 | 51 | 2.54 | 54 | 2.56 | 53 | 2.45 | 56 | 2.61 |
| H | 76 | 3.72 | 76 | 3.78 | 75 | 3.73 | 77 | 3.65 | 75 | 3.47 | 75 | 3.50 |
| I | 44 | 2.15 | 45 | 2.24 | 46 | 2.29 | 48 | 2.28 | 47 | 2.17 | 47 | 2.19 |
| P | 102 | 5.00 | 99 | 4.93 | 112 | 5.57 | 108 | 5.12 | 114 | 5.28 | 115 | 5.36 |
| Q | 14 | 0.69 | 18 | 0.90 | 12 | 0.60 | 17 | 0.81 | 17 | 0.79 | 17 | 0.79 |
| **Poorly characterized** | | | | | | | | | | | | |
| R | 128 | 6.27 | 136 | 6.77 | 139 | 6.91 | 141 | 6.69 | 146 | 6.76 | 147 | 6.86 |
| S | **338** | **16.55^b^** | **345** | **17.18^b^** | **327** | **16.26^b^** | **362** | **17.16^b^** | **370** | **17.12^b^** | **364** | **16.98^b^** |

^a^ J: translation, ribosomal structure and biogenesis; A: RNA processing and modification; K: transcription; L: replication, recombination and repair; B: chromatin structure and dynamics; D: cell cycle control, cell division, chromosome partitioning; V: defense mechanisms; T: signal transduction mechanisms; M: cell wall/membrane/envelope biogenesis; N: cell motility; U: intracellular trafficking, secretion, and vesicular transport; O: posttranslational modification, protein turnover, chaperones; C: energy production and conversion; G: carbohydrate transport and metabolism; E: amino acid transport and metabolism; F: nucleotide transport and metabolism; H: coenzyme transport and metabolism; I: lipid transport and metabolism; P: inorganic ion transport and metabolism; Q: secondary metabolites biosynthesis, transport and catabolism; R: general function prediction only; S: function unknown.

^b^ The four most abundant functional categories within the chromosomes of *A. caldus* strains.
