# Supplementary material for: Gene Turnover Contributes to the Evolutionary Adaptation of Acidithiobacillus caldus: Insights from Comparative Genomics
Source: Front Microbiol. 2016 Dec 6;7:1960. doi: 10.3389/fmicb.2016.01960 (PMC5138436; doi:10.3389/fmicb.2016.01960)
Supplement: Supplementary file 3 [file Table_3.docx]

**Supplementary Table S3** Statistics of transposases within accessory genes of *A. caldus* strains.

| **Strain** | **Number of transposase^a^** |
| --- | --- |
| SM-1 | 87 |
| ATCC 51756 | 68 |
| S1 | 11 |
| DX | 23 |
| ZBY | 34 |
| ZJ | 26 |

^a^ The putative transposases in each strain were predicted by aligning against the NCBI-nr database.
