# Supplementary material for: Gene Turnover Contributes to the Evolutionary Adaptation of Acidithiobacillus caldus: Insights from Comparative Genomics
Source: Front Microbiol. 2016 Dec 6;7:1960. doi: 10.3389/fmicb.2016.01960 (PMC5138436; doi:10.3389/fmicb.2016.01960)
Supplement: Supplementary file 5 [file Table_5.docx]

**Supplementary Table S5** Classification of insert sequences (IS) families in the *A. caldus* strains.

| **IS Family** | **Number** | | | | | |
| --- | --- | --- | --- | --- | --- | --- |
|  | **SM-1** | **ATCC 51756** | **S1** | **DX** | **ZBY** | **ZJ** |
| IS1 | 2 | 2 | 1 | 0 | 0 | 0 |
| IS110 | 6 | 3 | 3 | 4 | 2 | 4 |
| IS1380 | 0 | 1 | 2 | 4 | 2 | 4 |
| IS1595 | 4 | **11^a^** | 1 | 2 | 4 | 2 |
| IS200/IS605 | 3 | 2 | 2 | 1 | 2 | 1 |
| IS21 | **16^a^** | 6 | **5^a^** | **9^a^** | **8^a^** | **10^a^** |
| IS256 | 8 | **21^a^** | **5^a^** | 3 | **7** | 4 |
| IS3 | 3 | 3 | 1 | 0 | 0 | 2 |
| IS4 | 2 | 2 | 2 | 2 | 2 | 3 |
| IS481 | 3 | 4 | 1 | 0 | 1 | 1 |
| IS5 | **32^a^** | **31^a^** | **6^a^** | **12^a^** | **18^a^** | **14^a^** |
| IS630 | 4 | 3 | 1 | 4 | 0 | 4 |
| IS66 | **24^a^** | **21^a^** | 4 | 3 | 3 | 3 |
| IS91 | 3 | 2 | 2 | 4 | 4 | 5 |
| ISKra4 | 1 | 0 | 0 | 0 | 0 | 0 |
| ISL3 | **42^a^** | 8 | **10^a^** | **13^a^** | **8^a^** | **13^a^** |
| ISNCY | 0 | 1 | 0 | 1 | 0 | 1 |
| Tn3 | 11 | 9 | **6^a^** | **7** | **7** | **8^a^** |
| Total | 164 | 130 | 52 | 69 | 68 | 79 |

^a^ The most abundant IS families in the chromosomes of *A. caldus* strains.
