# Supplementary material for: Gene Turnover Contributes to the Evolutionary Adaptation of Acidithiobacillus caldus: Insights from Comparative Genomics
Source: Front Microbiol. 2016 Dec 6;7:1960. doi: 10.3389/fmicb.2016.01960 (PMC5138436; doi:10.3389/fmicb.2016.01960)
Supplement: Supplementary file 6 [file Table_6.docx]

**Supplementary Table S6** Detailed annotation of putative genomic islands in the *A. caldus* strains.

| **Genomic islands ID** | **Island position (start → end)** | **Length (bp)** | **Number of CDS** | **GC(%)** |
| --- | --- | --- | --- | --- |
| ***A. caldus* SM-1^a^** | | | | |
| GI-1 | 183,883 **→** 192,469 | 8,586 | 9 | 56.11 |
| GI-2 | 229,914 **→** 234,889 | 4,975 | 5 | 51.46 |
| GI-3 | 404,183 **→** 415,860 | 11,677 | 11 | 65.76 |
| GI-4 | 515,260 **→** 519,644 | 4,384 | 8 | 55.82 |
| GI-5 | 523,346 **→** 529,176 | 5,830 | 5 | 52.35 |
| GI-6 | 555,813 **→** 560,538 | 4,725 | 6 | 53.19 |
| GI-7 | 742,727 **→** 748,808 | 6,081 | 6 | 52.71 |
| GI-8 | 748,743 **→** 761,270 | 12,527 | 16 | 60.85 |
| GI-9 | 753,136 **→** 758,153 | 5,017 | 7 | 59.28 |
| GI-10 | 768,173 **→** 772,648 | 4,475 | 3 | 50.84 |
| GI-11 | 780,116 **→** 787,809 | 7,693 | 5 | 55.83 |
| GI-12 | 894,349 **→** 898,768 | 4,419 | 9 | 57.98 |
| GI-13 | 1,013,904 **→** 1,019,985 | 6,081 | 4 | 52.89 |
| GI-14 | 1,032,865 **→** 1,039,074 | 6,209 | 5 | 53.07 |
| GI-15 | 1,039,894 **→** 1,045,089 | 5,195 | 5 | 49.20 |
| GI-16 | 1,141,700 **→** 1,146,804 | 5,104 | 5 | 52.98 |
| GI-17 | 1,214,068 **→** 1,272,364 | 58,296 | 52 | 59.63 |
| GI-18 | 1,298,007 **→** 1,302,296 | 4,289 | 6 | 52.11 |
| GI-19 | 1,629,234 **→** 1,649,170 | 19,936 | 16 | 55.03 |
| GI-20 | 1,638,581 **→** 1,643,666 | 5,085 | 5 | 52.09 |
| GI-21 | 1,864,812 **→** 1,870,388 | 5,576 | 10 | 51.81 |
| GI-22 | 1,895,609 **→** 1,900,213 | 4,604 | 7 | 58.01 |
| GI-23 | 1,944,359 **→** 1,949,039 | 4,680 | 8 | 54.68 |
| GI-24 | 1,955,427 **→** 1,960,152 | 4,725 | 6 | 53.21 |
| GI-25 | 2,053,838 **→** 2,061,445 | 7,607 | 8 | 54.52 |
| GI-26 | 2,084,070 **→** 2,103,306 | 19,236 | 18 | 55.83 |
| GI-27 | 2,099,970 **→** 2,105,253 | 5,283 | 7 | 53.97 |
| GI-28 | 2,113,657 **→** 2,118,444 | 4,787 | 5 | 51.37 |
| GI-29 | 2,122,415 **→** 2,127,098 | 4,683 | 5 | 54.15 |
| GI-30 | 2,134,091 **→** 2,139,322 | 5,231 | 8 | 57.33 |
| GI-31 | 2,784,913 **→** 2,803,241 | 18,328 | 22 | 59.69 |
| ***A. caldus* ATCC 51756^b^** | | | | |
| GI-1 | 170,500 **→** 220,099 | 49,600 | 56 | 56.33 |
| GI-2 | 235,000 **→** 252,599 | 17,600 | 18 | 55.04 |
| GI-3 | 1,004,500 **→** 1,0330,99 | 28,600 | 25 | 56.56 |
| GI-4 | 1,339,000 **→** 1,375,599 | 36,600 | 28 | 56.24 |
| GI-5 | 1,606,500 **→** 1,636,099 | 29,600 | 28 | 54.69 |
| GI-6 | 1,646,000 **→** 1,675,099 | 29,100 | 33 | 56.16 |
| GI-7 | 1,753,000 **→** 1,770,599 | 17,600 | 23 | 56.03 |
| GI-8 | 1,775,500 **→** 1,793,099 | 17,600 | 18 | 55.77 |
| GI-9 | 1,903,500 **→** 1,975,099 | 71,600 | 70 | 54.02 |
| GI-10 | 2,107,500 **→** 2,132,099 | 24,600 | 24 | 57.70 |
| GI-11 | 2,296,000 **→** 2,318,099 | 22,100 | 18 | 60.72 |
| ***A. caldus* DX^a^** | | | | |
| GI-1 | contig208: 46,243 **→** 46,243 | 10,277 | 10 | 65.75 |
| GI-2 | contig209: 13,525 → 14,010 -N- contig307: 1 → 2,528^c^ |  |  |  |
| GI-3 | contig350: 2,047 → 8,149 | 6,103 | 7 | 46.63 |
| GI-4 | contig20: 8,147 → 14,709 | 6,563 | 5 | 56.74 |
| GI-5 | contig92: 265 → 1 -N- contig323: 4,965 → 1,781^c^ |  |  |  |
| GI-6 | contig16: 3,788 → 1 -N- contig47: 12,219 → 9,026^c^ |  |  |  |
| GI-7 | contig29: 4,003 → 8,681 | 4,679 | 8 | 54.67 |
| GI-8 | contig29: 15,124 → 19,794 | 4,671 | 6 | 53.14 |
| GI-9 | contig61: 16,893 → 35,329 | 18,437 | 19 | 56.93 |
| GI-10 | contig61: 12,076 → 17,358 | 5,283 | 7 | 54.36 |
| GI-11 | contig376: 1,700 → 6,450 -N- contig51: 2,109 → 709^c^ |  |  |  |
| GI-12 | contig332: 954→ 6,047 | 5,094 | 5 | 59.25 |
| GI-13 | contig340: 6,008 → 8,625 -N- contig381: 1 → 14,758^c^ |  |  |  |
| ***A. caldus* ZBJ^a^** | | | | |
| GI-1 | contig279: 1→ 6,210 | 6,210 | 7 | 51.84 |
| GI-2 | contig333: 144 → 2,352 -N- contig352: 4,303 → 3,479^c^ |  |  |  |
| GI-3 | contig220: 4,265→ 13,035 | 4,468 | 5 | 51.39 |
| GI-4 | contig149: 4,858 → 1 -N- contig264: 1 → 7,412^c^ |  |  |  |
| GI-5 | contig233: 4,598→ 14,874 | 10,277 | 10 | 65.75 |
| GI-6 | contig81: 8,269→ 14,831 | 6,563 | 5 | 56.74 |
| GI-7 | contig26: 2,761 → 1 -N- contig264: 16,758 → 15,364^c^ |  |  |  |
| GI-8 | contig229: 26,227→ 33,469 | 7,243 | 9 | 55.49 |
| GI-9 | contig3: 33,401 → 37,165 -N- contig171: 1 → 524^c^ |  |  |  |
| GI-10 | contig156: 37,499 → 39,798 -N- contig85: 891 → 20^c^ |  |  |  |
| GI-11 | contig7: 14,126→ 18,804 | 4,679 | 8 | 54.67 |
| GI-12 | contig7: 3,013→ 7,683 | 4671 | 6 | 53.14 |
| GI-13 | contig222: 423 → 1 -N- contig79: 1 → 3,236^c^ |  |  |  |
| GI-14 | contig1: 1,033 → 1,995 -N- contig275: 1 → 3,143^c^ |  |  |  |
| GI-15 | contig105: 6,712 → 11,994 | 5,283 | 7 | 54.36 |
| GI-16 | contig105: 1,385 → 1 -N- contig104: 5,567 → 3,317^c^ |  |  |  |
| GI-17 | contig363: 1,761 → 6,527 -N- contig94: 2,064 → 648^c^ |  |  |  |
| GI-18 | contig287: 20,938 → 22,117 -N- contig69: 1 →2,582^c^ |  |  |  |
| GI-19 | contig132: 2 → 4,342 | 4,341 | 6 | 55.19 |
| GI-20 | contig131: 665 → 10,584 | 9,920 | 9 | 52.84 |
| GI-21 | contig131: 20,108 → 24,391 -N- contig19: 1 → 2,985^c^ |  |  |  |
| GI-22 | contig96: 4,786 → 7,749 -N- contig339: 1 → 261^c^ |  |  |  |
| ***A. caldus* ZJ^a^** | | | | |
| GI-1 | contig198: 5,505 → 1 -N- contig197: 739 → 1^c^ |  |  |  |
| GI-2 | contig35: 8,602 → 8,874 -N- contig365: 1 → 16,051^c^ |  |  |  |
| GI-3 | contig152: 202 → 4,669 | 4,468 | 5 | 51.39 |
| GI-4 | contig363: 2,047 → 8,149 | 6,103 | 7 | 46.63 |
| GI-5 | contig99: 17,524 → 27,800 | 10,277 | 10 | 65.75 |
| GI-6 | contig244: 265 → 1 -N- contig334: 4,965 → 1,781^c^ |  |  |  |
| GI-7 | contig259: 2 → 4,460 | 4,459 | 5 | 59.07 |
| GI-8 | contig259: 13,215 → 16,963 -N- contig23: 1 → 1,466^c^ |  |  |  |
| GI-9 | contig113: 180 → 1 -N- contig171: 4,565 → 1^c^ |  |  |  |
| GI-10 | contig378: 11,476 → 16,154 | 4,679 | 8 | 54.67 |
| GI-11 | contig231: 16,725 → 17,745 -N- contig276: 7,111 → 3,924^c^ |  |  |  |
| GI-12 | contig132: 14,024 → 35,329 | 21,306 | 23 | 56.50 |
| GI-13 | contig132: 12,077 → 17,359 | 5,283 | 7 | 54.36 |
| GI-14 | contig132: 1 → 6,820 | 6,820 | 11 | 53.45 |
| GI-15 | contig340: 1,745 → 6,495 -N- contig59: 1 → 1,401^c^ |  |  |  |
| GI-16 | contig53: 1,253 → 6,346 | 5,094 | 5 | 59.27 |
| GI-17 | contig249: 14,316 → 19,873 | 5,558 | 7 | 57.97 |
| GI-18 | contig131: 2 → 347 -N- contig27: 1 → 2,946^c^ |  |  |  |
| GI-19 | contig27: 1,673 → 7,971 | 6,299 | 8 | 60.06 |
| GI-20 | contig205: 2 → 17,878 | 17,877 | 15 | 52.51 |
| GI-21 | contig205: 24,800 → 36,148 | 11,349 | 14 | 54.36 |

^a^ These Genomic islands were predicted using IslandViewer 3 with at least one method.

^b^ Horizontally transferred elements were predicted using SeqWord Genomic Island Sniffer.

^c^ The letter ‘N’ means the gap between contigs. For these draft genomes, only genomic island-related sequences with a gap of less than 2 were listed in this table. Additionally, the putative GI sequences with gap were not further calculated the number of ORF as well as GC content.
